# Optimum radiation source for radiation therapy of skin cancer

**DOI:** 10.1120/jacmp.v16i5.5407

**Published:** 2015-09-08

**Authors:** Habib Safigholi, William Y. Song, Ali S. Meigooni

**Affiliations:** ^1^ Department of Medical Physics Odette Cancer Centre, Sunnybrook Research Institute, Sunnybrook Health Sciences Centre, University of Toronto Toronto ON Canada; ^2^ Department of Radiation Therapy Comprehensive Cancer Centers of Nevada Las Vegas NV USA; ^3^ Department of Health Physics and Diagnostic Science School of Allied Health Science, University of Nevada Las Vegas (UNLV) Las Vegas NV USA

**Keywords:** optimum radiation source, skin cancer, brachytherapy, electron beam therapy, electronic brachytherapy

## Abstract

Several different applicators have been designed for treatment of skin cancers, such as scalp, hand, and legs using Ir‐192 HDR brachytherapy sources (IR‐HDRS), miniature electronic brachytherapy sources (eBT), and external electron beam radiation therapy (EEBRT). Although, all of these methodologies may deliver the desired radiation dose to the skin, but the dose to the underlying bone may become the limiting factor for selection of the optimum treatment technique. In this project, dose to the underlying bone has been evaluated as a function of the radiation type, thickness of the bone, and thickness of the soft tissue on top of bone, assuming the same radiation dose delivery to the skin. These evaluations are performed using Monte Carlo (MC) simulation technique with MCNP5 code. The results of these investigations indicate that, for delivery of the same skin dose with a 50 keV eBT, 4 MeV or 6 MeV EEBRT techniques, the average doses received by the underlying bones are 5.31, 2, or 1.75 times the dose received from IR‐HDRS technique, respectively. These investigations indicate that, for the treatment of skin cancer condition with bone immediately beneath skin, the eBT technique may not be the most suitable technique, as it may lead to excessive bone dose relative to IR‐HDRS and 6 MeV or 4 MeV electron beams.

PACS number: 87.53.Jw, 87.55.K‐

## I. INTRODUCTION

There are three major types of skin cancer: basal, squamous cell carcinoma, and melanoma. The first two (nonmelanoma) are occurring on the outer layer of skin (epidermis).^1^, ^2^, ^3^ Melanoma occurs on the skin at the palm of hands and soles of feet, scalp, ears, nails, and back, and may turn the skin color to black (melanin). In 2015, approximately 74,000 Americans will be diagnosed with stage I‐IV melanoma, which led approximately to 10,000 deaths.^(1)^ Nonmelanoma skin cancer affects approximately two to three million people each year in the United States.^(3)^ However, that has a lower mortality rate (less than 1000) and it significantly affects the quality of life.^(2)^ Treatment options for skin cancers include radiation therapy, surgery, chemotherapy, and photodynamic therapy. In radiation therapy, X‐rays or electron beams may be used to treat local skin cancers. A variety of radiation therapy techniques are used including superficial X‐rays,^(4)^ orthovoltage X‐rays,^(5)^ megavoltage photons,^(4)^ electron beam therapy,^(4)^ and brachytherapy.^2^, ^3^, ^6^, ^7^ Several groups have shown different percentages of tumor control results for nonmelanoma skin cancer treatment using superficial X‐rays with 93%–100%,^(4)^ orthovoltage X‐rays with 87%,^(8)^ electron beams with 72%–88%,^(4)^ isotope‐based HDR brachytherapy with 92%–98%,^(6)^ and 100% for eBT up to 2013.^(6)^

The physical differences between these types of skin radiation are shown by comparison of their percentage depth doses and dose profiles. Normally, the percentage depth‐dose curves of electron beams, with a field size of greater than the practical range of the electrons, have sharp falloffs beyond the depth of the maximum dose. This property is beneficial in electron beam therapy for sparing the tissues beyond the range of the electron beams. Conventionally, the rate of the energy loss in water or water‐equivalent materials for an electron beam is about 2 MeV/cm. However, for a superficial X‐ray, dose falloff is exponential with the maximum dose being at the skin level. Generally for external electron beam therapy, a protocol with about 40 Gy to 60 Gy total dose is delivered at the rate of 2 Gy to 3 Gy per fractions within four to six weeks.^4^, ^6^ However, for brachytherapy, skin treatment by IR‐HDRs and eBT, the patient receives 6 to 8 fractions for a total dose ranging from 30 Gy to 40 Gy in two weeks.^3^, ^7^, ^9^, ^10^, ^11^ Normally, all of these treatments are performed assuming homogenous tissue condition and ignoring the underlying bone.

Presently, there are three different eBT systems commercially available for clinical applications. These systems include the Axxent by Xoft Inc. (Fremont, CA),^12^, ^13^, ^14^ the Intrabeam Photon Radiosurgery Device by Carl Zeiss Surgical (Oberkochen, Germany),^12^, ^14^ and the Esteya by Elekta (Esteya EBS, Elekta AB‐Nucletron, Stockholm, Sweden).^(15)^ The main component of these systems is a miniature X‐ray tube that produces the bremsstrahlung radiation using electron energies ranging from 20 to 70 keV. Treatment of skin cancer by the eBT systems can be performed using the conical applicators that have been developed by the manufacturer.^3^, ^7^ Four eBT applicator sizes are available with diameters of 1 cm, 2 cm, 3.5 cm, and 5 cm. The nominal eBT SSD values are fixed to be 2 cm for the 1 cm, 2 cm, and 3.5 cm cones, and at 3 cm for the 5 cm cone.^(3)^

Skin therapy by IR‐HDRS technique can be performed using the Valencia and Leipzig applicators developed by Nucletron (Nucletron Corp., Veenendaal, The Netherlands),^(10)^ or the surface applicator developed by Varian (Varian Medical Systems, Palo Alto, CA).^(11)^ For Valencia and Leipzig applicators, diameters are 1 cm, 2 cm, and 3 cm and the nominal source to surface distance (SSD) is 1.5 cm (10, 11). For the Varian surface applicators, the field diameters range from 3 cm to 4.5 cm with nominal SSD of 1.25 cm.^(11)^

Electron beam therapy is performed using linear accelerators. There are different applicators which are used for skin EEBRT. The inner diameters of the applicators are ranging from 1 cm to 8 cm, with different bevel angles for treatment of local skin cancers.^(16)^

**Figure 1:**
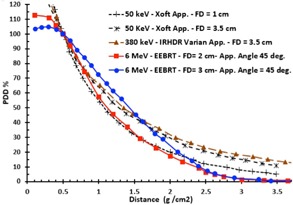
A comparison of the PDD for 6 MeV EEBRT with field diameter (FD) of 2 cm, and 3 cm for SSD=100 cm with applicator bevel angle of 45°,^(16)^ and PDDs of Xoft applicators and IR‐HDR Varian applicators with FD of 3.5 cm and 1 cm in water^(17)^ after renormalization to the same depth of 5 mm.

Fulkerson et al.^(17)^ published the percentage depth‐dose (PDD) data of the Varian IR‐HDR brachytherapy applicator with Ir‐GammaMedplus iX source and for 50 keV eBT Xoft applicators in homogenous phantom materials. These results were normalized to a depth of 5 mm and 3 mm, for IR‐HDR and eBT systems, respectively. As shown in Fig. 1, in the present study, the published datasets were renormalized to the same depth (i.e., 5 mm) for a direct comparison between eBT and IR‐HDRS systems. Moreover, this figure shows the %PDD of 6 MeV EEBRT, delivered using the Siemens PRIMUS linear accelerator (Siemens AG, Erlangen, Germany),^(16)^ for small field diameter (FD) of 2 cm and 3 cm, with 45° beveled applicators. These results indicate that %PDD of these techniques in homogenous treatment conditions are nearly the same, and one could choose any of them for the treatment of the skin cancer. However, these results do not provide any information for the behavior of these radiation sources under heterogeneous condition, particularly when the treatment volume contains bone immediately beneath the skin. Several other publications presented the dose distribution in different heterogeneous body structures, except skin cancer, when treated with IR‐HDR or eBT and low energy sources.^18^, ^19^, ^20^

The goal of this project is to investigate the dose to the underlying bone structure during the skin radiation therapy with eBT, IR‐HDRS or EEBRT treatment techniques. These evaluations are performed using Monte Carlo (MC) simulation technique. Dose to bone has been evaluated as a function of the radiation source, thickness of the bone, and the depth of the bone in the soft tissue.

## II. MATERIALS AND METHODS

MC simulations were performed using MCNP5 code.^(21)^ In these simulations, the monoenergetic photon beams of 10 keV, 15 keV, 20 keV, 30 keV, 50 keV, and 70 keV for covering the energy range of eBT, 380 keV photons for IR‐HDRS, and 4 MeV and 6 MeV monoenergetic electron beam for EEBRT were modeled. Moreover this work has been benchmarked with actual eBT spectrum, according our previous published results for 30 keV and 50 keV.^14^, ^20^, ^22^

**Figure 2:**
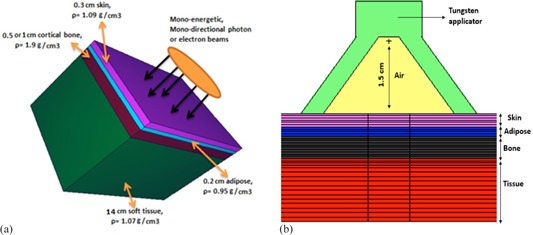
Schematic diagram of the heterogeneous phantom geometry used in Monte Carlo simulation in this project. This phantom shows skin, adipose, cortical bone, and soft tissue layers. The dimension of the cubic phantom are 15 cm×15 cm×15 cm. (a) The radiation field is assumed to be 2 cm diameter circular and parallel; (b) the radiation point source with Tungsten applicator by opening angle of 75° and 1.5 cm SSD.^(10)^

Figure 2(a) shows the schematic diagram of the simulated heterogeneous phantom. A 0.5 cm thick soft tissue (0.3 cm skin and 0.2 cm adipose) with underlying 0.5 cm or 1 cm cortical bone, followed by 14 cm soft tissue are utilized for simulations. Moreover, this evaluation was repeated for a 1.5 cm thick overlaying soft tissue (0.3 cm skin, 0.2 cm adipose, and 1 cm soft tissue), followed by 0.5 cm bone and 13 cm soft tissue. The phantom arrangements and dimensions selected here closely replicate the skin treatment of the sites such as scalp, forehead, knee, hand, feet, ear, and back along spine or over ribs. The photon or electron beams are considered to be perpendicular to the skin. The field size diameter was assumed to be 2 cm at surface for skin cancer treatments. For treatment of skin lesions with 6 MeV electrons, an applicator with inner diameters ranging from 1 cm to 8 cm is commonly used with bolus material to obviate the skin sparing effect of low‐energy electrons. However, in this research, for consistency with the IR‐HDR and eBT techniques, an applicator with similar dimension (i.e., 2 cm diameter) and field size was simulated for electrons. It is known that, for electron beams, a very small field size degrade %PDD distribution and shift the dose maximum depth to shallower depths. Each slab in the phantom is divided into 0.5 to 1 mm scoring thicknesses with 1 mm diameter, which are small enough to record the variation of the dose (F6 (MeV/g/source‐particle)) along the central axis of the beams. The F6 tally estimates the track length of the energy deposition.^(21)^ The MCNP5 default photon and electron cross section libraries, MCPLIB04 (04p) and el03 (03e), were applied for calculations, respectively. In this project, the calculations were performed with up to $10^{8}$ histories in order to produce the statistical uncertainties of less than 1% for all cases. The MCNP calculations for low‐energy photons were performed in the photon mode and for IR‐HDRS and EEBRT in the photon and electron mode. For all cases, dose to materials were scored. The overall geometry of the homogenous phantom is like the heterogeneous phantom, but constructed from the homogenous skin tissue type. To compare the bone‐sparing effect for eBT, low‐energy photons, IR‐HDRS, and EEBRT techniques, two different definitions have been used throughout the text as “normalized dose” and “dose ratio” defined as:

(1) $$Normalized\;Dose{\left(r\right)}_{H\;or\;Het}=\frac{D(r,tissue_{H\;or\;Het})}{D_{\text{max}}(tissue_{H})}$$

(2) $$Dose\;Ratio\;(r)=\frac{D(r,tissue_{Het})}{D(r,tissue_{H})}$$

where $D(r,\;tissue_{H}\;or_{Het}),D(r,\;tissue_{H})$, and $D(r,\;tissue_{Het})$ represent the dose at the depth “r” in homogeneous and/or heterogeneous phantom, and $D_{max}(tissue_{H})$ is the maximum dose value in homogeneous phantom.

In order to be able to compare the dose data by an actual applicator for eBT and IR‐HDRS, calculations were repeated with a same geometry shown in Fig. 2(b). In these calculations, simulations were performed for homogenous and heterogeneous phantoms using a Leipzig applicator with opening cone angle of 75° and 1.5 cm SSD, and radiation point sources.^(10)^ This approach is applicable with other applicators.

## III. RESULTS & DISCUSSION

**Figure 3:**
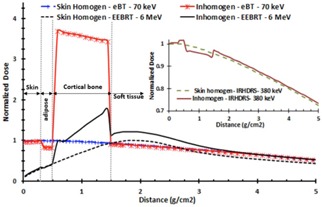
Dose comparison of the 70 keV photons, 6 MeV electrons, and 380 keV Ir‐192 for skin homogenous and inhomogeneous phantoms. All data were normalized to the maximum dose value in the skin homogeneous phantom. The bone thickness is 0.5 cm, according to Fig. 2(a).The graph for IR‐HDRS is drawn separately in the upper right corner of the figure to avoid the crowded curves.

Our results for normalized MC simulated dose distributions as a function of distance ($g/{\text{cm}}^{2}$) for skin treatment, assuming homogenous and heterogeneous conditions, for 70 keV eBT, 6 MeV EEBRT, and 380 keV IR‐HDRS, for 0.5 cm bone thickness are shown in Fig. 3. These results indicate that the normalized dose values to the skin layer for 70 keV photons in homogenous and heterogeneous conditions are nearly the same. However, in the adipose layer, dose value in the heterogeneous medium is approximately 17% lower (i.e., normalized dose=0.83) than the skin dose. Moreover, for this energy level, the normalized dose to the cortical bone layer is about 3.6 times larger than the skin dose. These differences are due to the variation in chemical compositions and density of different tissue types, especially at the low‐energy level where the photoelectric interaction occurs. Table 1 shows that the values of the mass absorption coefficient of the adipose and bone relative to skin for 70 keV are 0.849 and 3.713, respectively. These corresponding values are in excellent agreement with the 0.83 and 3.6 of the normalized dose for adipose and bone, respectively. For 6 MeV electrons, in the bone layer, the normalized dose is about 80% higher than the skin dose in the homogenous medium (i.e., normalized dose=0.83). This is due to the multiple Coulomb scattering in bone region and differences in mass stopping power. After the bone thickness, immediately there is a small increase in dose to soft tissue due to increasing scattering of electrons. Figure 3 clearly shows that there are fewer differences between soft tissue and bone dose for 6 MeV electron beam than 70 keV photons. However, still there are significantly higher doses to the bone than the skin for both of these two techniques.

For 380 keV IR‐HDRS, the normalized dose to adipose and bone in heterogeneous conditions are 1.8% larger and 2.8% smaller than the homogenous skin dose, respectively. This is due to Compton interaction range, which is independent of atomic number of the materials and is dependent on the electron density, which is nearly the same for all tissue. Moreover, the mass absorption coefficient of the tissue relative to skin is well matched to unity, according to the data in Table 1

**Figure 4:**
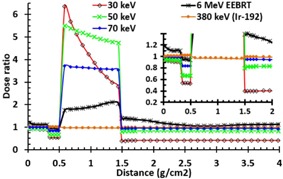
The dose ratio values in the inhomogeneous to the homogeneous phantoms vs. distance ($g/{\text{cm}}^{2}$) for the 30, 50, and 70 keV photons of the eBT, 380 keV IRHDRS, and 6 MeV EEBRT. The bone thickness is 0.5 cm according to Fig. 2(a). For the distance from 0 to $2\;g/{\text{cm}}^{2}$, the figure is zoomed in the upper corner of the graph.

Figure 4 shows a comparison between the dose ratio values as a function of depth in a heterogeneous geometry (Fig. 2(a)) for different radiation sources. These results indicate that for 30, 50, and 70 keV photons, average dose ratios (Eq. (2)) in skin regions are 0.9, 0.94, and 0.98, respectively. This is because of the impact of surface dose reduction due to the loss of backscatter from the bone interface. The published results indicated a surface dose reduction of 1% to 10% when the thickness of water‐equivalent material on top of a 1 cm thick bone was decreased from 20 mm to 0.5 mm by 33 keV or 50 keV beams, respectively.^5^, ^23^ Moreover, Fig. 4 indicates that the values of dose ratios for cortical bone layer are greater than the skin dose by factors of 4.24, 5.31, and 3.62, respectively. These dose ratios are consistence with the mass absorption coefficient ratio of adipose and bone over skin in Table 1 for 30, 50, and 70 keV, respectively. These results demonstrate that the dose ratios for skin and adipose layers for 380 keV IR‐HDRS are approximately unity. However for bone layer, the ratio is 0.972. For 6 MeV EEBRT, the dose ratio to the skin in heterogeneous medium is approximately 1.5% larger than the homogenous condition. This increase of the dose is attributed to the backscattering radiation from bone. Dose ratios for the bone and soft tissue layers for 6 MeV EEBRT are 1.75 and 1.15, respectively.

**Table 1:** Mass absorption coefficient of adipose, bone, and soft tissue over skin for keV energy range. These values are calculated from the elemental composition of the NIST data for any materials.^(24)^

*Energy (KeV)*	$\left(\frac{\mu_{en}}{\rho }\right)$ *adipose skin*	$\left(\frac{\mu_{en}}{\rho }\right)$ *bone skin*	$\left(\frac{\mu_{en}}{\rho }\right)$ *Soft tissue skin*
10	0.667	6.088	1.133
15	0.659	6.820	1.140
20	0.657	7.275	1.144
30	0.673	7.589	1.146
50	0.771	5.840	1.090
70	0.849	3.713	1.067
100	0.973	1.834	1.018
380	1.016	1.052	1.003

**Figure 5:**
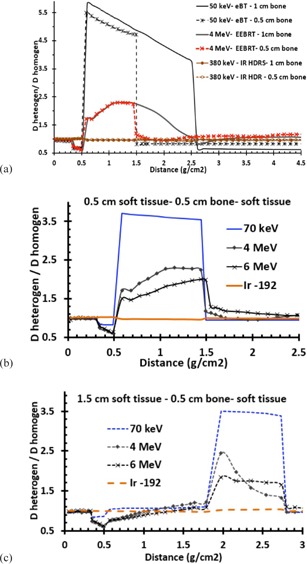
Comparison (a) of the fixed soft tissue geometry before and after of the 0.5 and 1 cm cortical bone thicknesses. The tissue before the bone was 0.3 cm skin with 0.2 cm adipose layers. The bone thicknesses are followed by the soft tissue layer. Dose ratio (b) for (0.3 cm skin+0.2 cm adipose+0.5 cm bone+soft tissue) for different beams. Dose ratio (c) for (0.3 cm skin+0.2 cm adipose+1 cm soft tissue+0.5 cm bone+soft tissue) for different beams.

Figure 5(a) shows the comparison of the dose ratio for 0.5 and 1 cm cortical bone located at 0.5 cm depth. The results are shown for 50 keV eBT, 380 keV IR‐HDR, and 4 MeV EEBRT. The behaviors of the 0.5 cm and 1 cm bone thicknesses are very similar. The major differences are on the dose to the underlying soft tissue. A comparison of Fig. 5(b) with Fig. 5(c) shows that, for the three treatment modalities, the dose ratio to 0.5 cm thick bone for a 0.5 cm overlaying tissue is very similar to that one with 1.5 cm overlaying tissue. In addition, these figures show that the 4 MeV and 6 MeV electron beams produces lower bone dose ratio compared to the eBT beams. These results indicate that, for 70 keV photon beams, the average dose ratio for 0.5 cm bone with 0.5 cm and 1.5 cm soft tissue are 3.62 and 3.45, respectively. The corresponding values for 4 MeV electron beams were 2.1 and 1.74, and for 6 MeV electron beams were 1.73 and 1.71, respectively. The published results for bone and mucosal doses at oral and nasal cavity phantom in skin cavity, by the 150 and 220 kVp photons (corresponding to 50 keV and 73.3 keV monoenergetic beams) for the 0.2–1 cm spongy bone which is supported by the 0.5–2 cm water thickness on top of the bone, created a factor of bone mean doses range from 4.5 to 1.5, respectively.^(23)^ The differences here with the study by Chow and Jiang^(23)^ are due to the difference between cortical and spongy bones and beam facilities. However, for spongy bone, such as sternum and the bone between oral or nasal cavity, the electron densities or CET are not much different from water or soft tissue for 4 or 6 MeV electrons.

Table 2 presents the comparison between dose values to bone as a function of beam energy and beam type, for a delivery of 5000 cGy to skin. These results indicate that the underlying bone will receive a 5 times (or more) larger dose from 10 keV, 15 keV, 20 keV, 30 keV, 50 keV, 70 keV of eBT than IR‐HDRS treatment. However, the absorbed doses to bone from 4 MeV and 6 MeV EEBRT techniques are approximately 2.14 and 1.79 times the dose from than IR‐HDRS treatment. To verify the impact of the beam divergence from a real applicator on these results, simulations were performed for homogenous and inhomogeneous phantoms using a sample applicator — with opening cone angle of 75° and 1.5 cm SSD, and radiation point sources — that was noted in publication by Niu et al.^(10)^ This applicator was the same as eBT applicator. The results in Table 2 indicate that the dose to bone with real cone applicator is not significantly different than the nondivergent beam (i.e., differences are less than 3%).

Finally, the actual spectrum eBT were used instead of the monoenergetic spectrum for the benchmarking process. We evaluated the spectrum of eBT in our previous works.^14^, ^20^, ^22^ The results show that the maximum differences of 30 keV and 50 keV eBT spectra for dose ratio with 0.5 cm bone were less than 8% and 5.5%, respectively. The differences between monoenergetic and full spectrum were not found to be significant. The reason is that the effects of different components (such as source geometry and filter) were considered (and consequently removed) in the dose ratios, as showed in (1), (2).

**Table 2:** Dose values to underlying bone for delivery of 5000 cGy to skin surface for different treatment techniques. These results were obtained for the 0.5 cm soft tissue (0.3 cm skin and 0.2 cm adipose) on top of the 0.5 cm thick bone, followed by 14 cm soft tissue

*Different Techniques*	*Dose, Nondivergent Beam (cGy)*	*Dose, Cone Applicator (cGy)*
4 MeV EEBRT	10,450	–
6 MeV EEBRT	8,735	–
380 keV IR‐HDRS	4,875	5,000
10 keV eBT	19,250	19,290
15 keV eBT	26,750	26,730
20 keV eBT	30,270	30,870
30 keV eBT	31,750	33,700
50 keV eBT	27,450	28,900
70 keV eBT	18,550	20,250

## IV. CONCLUSIONS

In the radiation therapy of skin cancers such as scalp, forehead, knee, hand, legs, ear, and back (along the spine) where the underlying bone is located at the close vicinity of the treatment target, selection of the source of radiation is very critical. It seems that, for these treatments, the uses of low‐energy photons from eBT sources are desirable due to the minimal room shielding requirements. In addition, this technique does not include any radioactive element, and it is easier to possess in term of the regulatory requirements. Clinical benefit of this system has been discussed by various investigators, and the dosimetric characteristics of eBT have been published in different scientific journals.^12^, ^13^, ^14^, ^15^ However, all of these publications indicate that eBT is an excellent technique for treatment of skin cancers in homogeneous condition. The results of our investigations indicate that, for the treatment of skin cancer with underlying bone, the eBT technique may not be the most suitable technique as it may deliver excessive bone dose relative to other treatment modalities, such as IR‐HDRS and 4 MeV or 6 MeV electron beams.

## ACKNOWLEDGMENTS

The authors would like to present their appreciation to Dr. Andrew Cohen and Dr. Courtney Knaup from Comprehensive Cancer Centers of Nevada for their invaluable editorial work on this manuscript prior to its submission. We deeply thank Dr. Jose Perez‐Calatayud, Dr. Facundo Ballaster for information of the Esteya source, and Dr. Mark J. Rivard for providing mass attenuation coefficients.
